# A^3^-Coupling catalyzed by robust Au nanoparticles covalently bonded to HS-functionalized cellulose nanocrystalline films

**DOI:** 10.3762/bjoc.9.155

**Published:** 2013-07-10

**Authors:** Jian-Lin Huang, Derek G Gray, Chao-Jun Li

**Affiliations:** 1 Department of Chemistry, McGill University, 801 Sherbrooke Street West, Montreal, Quebec H3A0B8, Canada

**Keywords:** A^3^-coupling reaction, cellulose nanocrystallites (CNCs) films, gold catalysis, water or without solvent

## Abstract

We decorated HS-functionalized cellulose nanocrystallite (CNC) films with monodisperse Au nanoparticles (AuNPs) to form a novel nanocomposite catalyst AuNPs@HS-CNC. The uniform, fine AuNPs were made by the reduction of HAuCl_4_ solution with thiol (HS-) group-functionalized CNC films. The AuNPs@HS-CNC nanocomposites were examined by X-ray photoelectron spectroscopy (XPS), TEM, ATR-IR and solid-state NMR. Characterizations suggested that the size of the AuNPs was about 2–3 nm and they were evenly distributed onto the surface of CNC films. Furthermore, the unique nanocomposite Au@HS-CNC catalyst displayed high catalytic efficiency in promoting three-component coupling of an aldehyde, an alkyne, and an amine (A^3^-coupling) either in water or without solvent. Most importantly, the catalyst could be used repetitively more than 11 times without significant deactivation. Our strategy also promotes the use of naturally renewable cellulose to prepare reusable nanocomposite catalysts for organic synthesis.

## Introduction

Organic synthesis is usually performed in organic solvents; however, from a green chemistry perspective, evaporation and discharge of organic solvents not only generates chemical waste but also causes environmental pollution [1–2]. In the past few decades, aqueous-phase organic reactions have achieved great success [3–5]. The classic examples include the Grignard-type reactions [6–7], transition-metal catalyzed C–C bond formations [8–9] and cross-dehydrogenative coupling (CDC) reactions [10–13]. In addition, the three-component aldehyde–alkyne–amine (A^3^) coupling and asymmetric aldehyde–alkyne–amine (AA^3^) coupling reactions have received increasing attention due to the easy formation of high-value product propargylamines [14–16]. Notably, the A^3^ coupling reaction has also been achieved in aqueous media or without solvent by gold catalysis [17–19]. However, up until now, most of the reactions are conducted by using homogenous organometallic catalysts. They usually show the high catalytic activity and selectivity [20]; however, homogeneous catalysts are difficult to adopt in large-scale industrial settings because of challenges associated with recovery and reuse of the catalysts from the reaction system, which may also increase the cost and cause environmental pollution by metallic ions. Heterogeneous catalysts could overcome the above problems [21]; however, they usually show lower catalytic activities compared with homogeneous catalysts, which may be caused by blocking the diffusion and adsorption of organic reactant molecules and products or the poor dispersion of active sites [22–23].

Cellulose nanocrystals (CNCs) have emerged as a new class of nanomaterials owing to their renewable, environmentally benign, naturally abundant, biodegradable and biocompatible nature, as well as their excellent mechanical properties and anticipated low cost [24–26]. CNCs are obtained from semicrystalline cellulose derived from wood fibers and plants. Potential applications for CNCs include nanocomposite formulation, polymer reinforcement, drug delivery [27], enzyme immobilization [28], biomedical applications [29] and as templates for the synthesis of nanomaterials [30]. The deposition of metal nanoparticles onto the surface of CNCs can lead to new nano-heterogeneous catalysts for organic synthesis. Recently, CNCs have been used as an effective support for Pd nanoparticles [31], AuNPs [32], SeNPs [33], NiNPs [34] and Au–Ag alloy NPs [35] for greener organic synthesis. However, to date, studies on combining the surface chemistry of CNCs with metal nanoparticles in catalysis are still very limited.

This paper describes the use of HS-functionalized CNCs decorated with gold nanoparticles as a novel class of heterogeneous catalysts for greener organic reactions. AuNPs were formed and deposited on the surface of HS-functionalized CNCs by coordination with the free HS-ligands. The as-prepared Au@HS-CNC catalyst displays high catalytic efficiency in A^3^-coupling reactions performed in either aqueous media or without solvent. More importantly, it can be used repetitively up to 11 times without significant loss of catalytic efficiency.

## Results and Discussion

### Preparation and characterizations of nanocomposite Au@HS-CNC catalyst

**Scheme 1:**
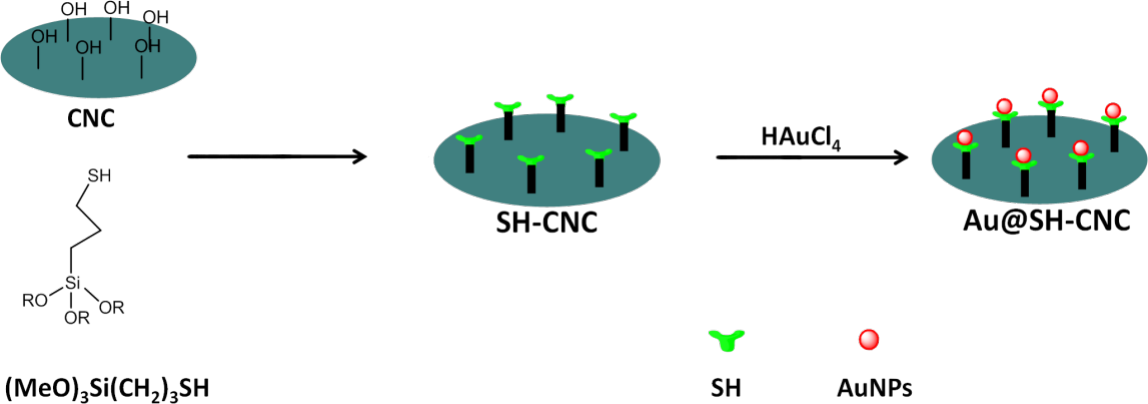
Sketch illustrating preparation of the Au@HS-CNC catalyst.

Scheme 1 briefly illustrates the preparation of the Au@HS-CNC catalyst. First, the HS-CNC composite was prepared by using a modified procedure reported by MacLachlan et al [30]. In a typical procedure, 30 mL of a 2.1% aqueous CNC suspension was sonicated for 10 min (see Methods for details of CNC preparation in Supporting Information File 1) and pH adjusted to 2.9 with AcOH. 3-Mercaptopropyltrimethoxysilane (1.0 mL, 4.5 mmol) was added to the CNC suspension and the mixture was stirred at 25 °C until a homogeneous mixture was obtained (typically about 4 h). This solution was cooled to room temperature, and then dried on a polystyrene Petri dish. After slow evaporation at room temperature, the nanocomposite films of the HS-CNC materials were dried at 120 °C for 2 h. Then, the films were successively soxhlet extracted with EtOH for 6 h and filtered. Finally, the HS-CNC films were added into 0.12 M HAuCl_4_ ethanol solution and kept under stirring at room temperature for 24 h (during this step, the Au^3+^ was reduced to Au^0^ by the HS-groups attached on CNC), then filtered and dried at 40 °C overnight. Alternatively, the Au@HS-CNC was also synthesized by using a modified procedure reported by Tingaut et al. Only the method of thiol functionalized CNC support (HS-CNC) is different from that reported by MacLachlan et al. (see Methods for the details about the catalyst preparation).

### Structure characterizations

**Figure 1:**
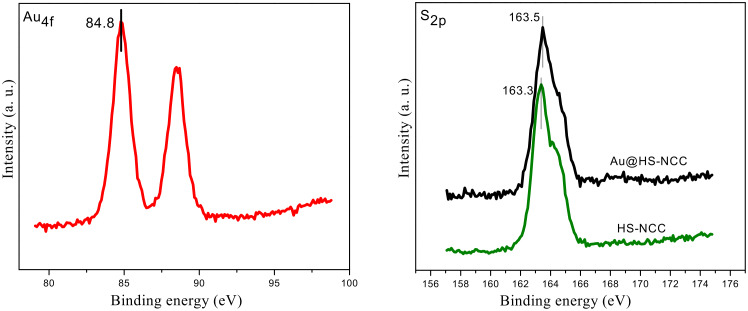
Au_4f_ and S_2p_ XPS spectra of the Au@HS-CNC (4.4 mol %) catalyst.

**Figure 2:**
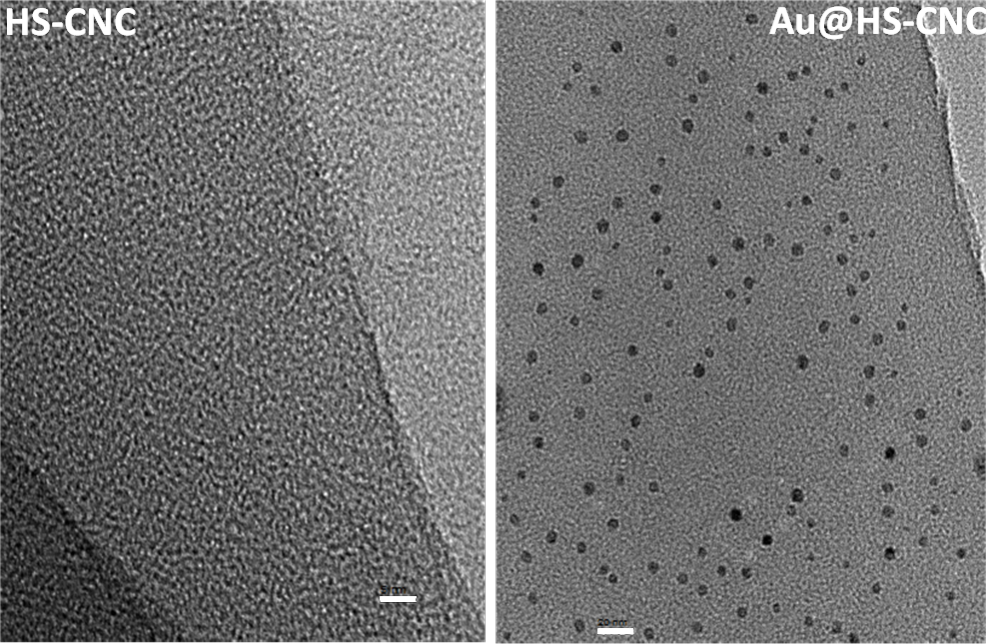
TEM pictures of the HS-NCC and Au@HS-CNC (4.4 mol %) catalyst (scale bar: 5 nm).

**Figure 3:**
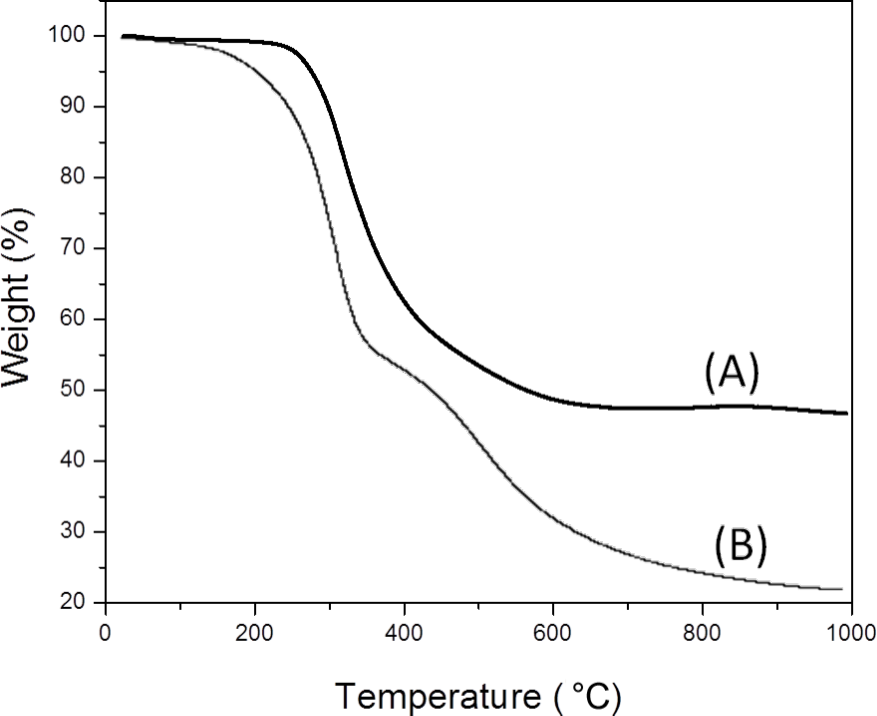
Thermogravimetric behavior of the Au@HS-CNC (4.4 mol %) catalyst (A) and CNC (B).

**Figure 4:**
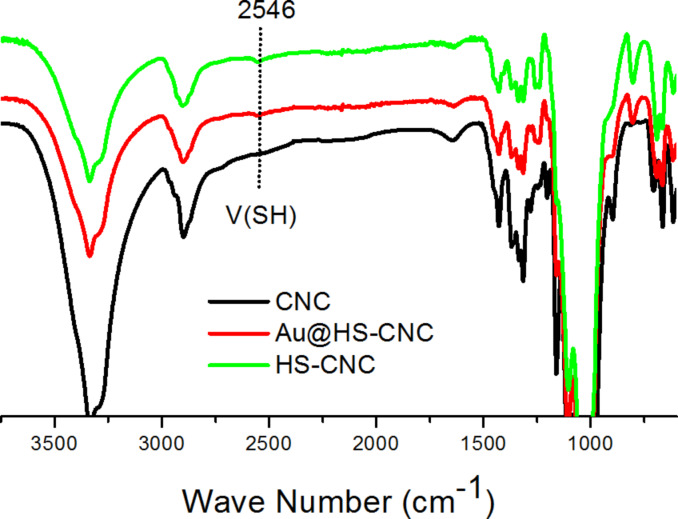
FT-IR spectra of CNC, HS-CNC, and Au@HS-CNC (4.4 mol %) catalyst.

**Figure 5:**
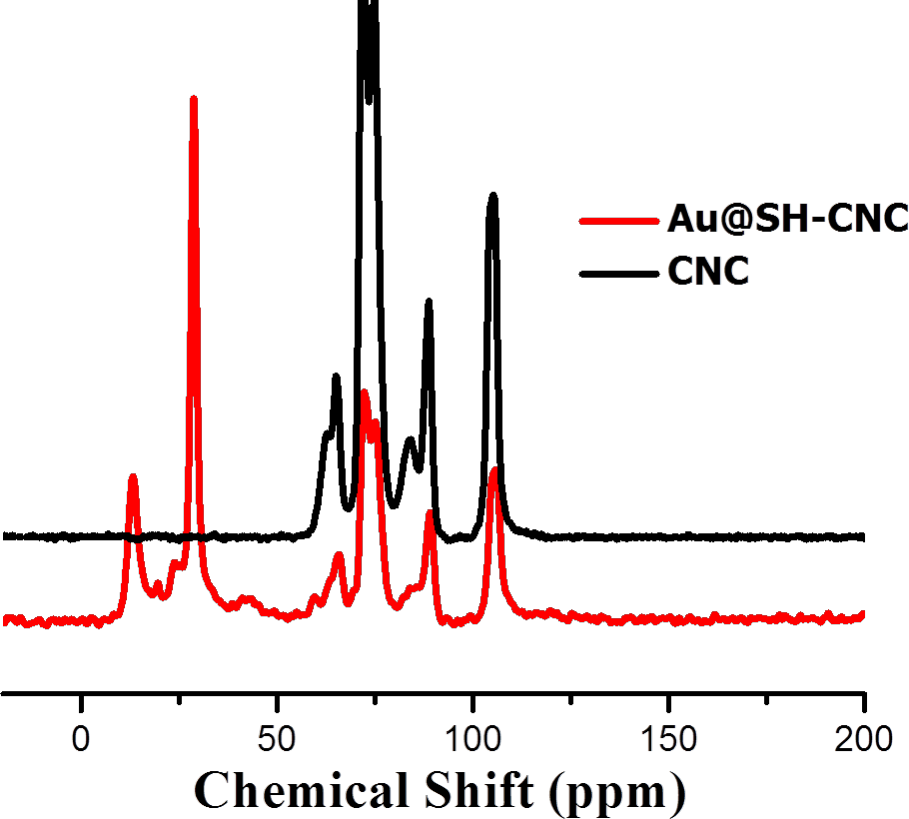
Solid-state ^13^C NMR spectra of the CNC and Au@HS-CNC (4.4 mol %) catalyst.

The XPS spectra (Figure 1) demonstrated that the binding energy of the Au species in the Au@HS-CNC (4.4 mol %) sample was 84.8 eV for the Au_4f7/2_ level corresponding to zero-valent Au, according to reference data reported by Li et al. [17], and no other peak was observed. This indicated that metal ions (Au^3+^) have been reduced to their metallic states (Au^0^). TEM pictures in Figure 2 further confirm this result. The HRTEM images in Figure S1 clearly show the size (2–3 nm) and lattice of the Au nanoparticles on the surface of the Au@HS-CNC (4.4 mol %) catalyst. The S species were mainly present in −2 states, corresponding to HS-groups with the binding energy around 163.5 in the S_2p_ level. Thermogravimetric analysis (Figure 3) showed that the deposition of AuNPs onto CNC apparently enhanced the thermal stability of the Au@HS-CNC (4.4 mol %) films, which might be due to a composite of the saline reagent (3-mercaptopropyltrimethoxysilane). The Au@HS-CNC (4.4 mol %) decomposed at above 250 °C under an inert atmosphere, making them an attractive catalyst for catalytic reactions. The FT-IR spectra of CNC, HS-CNC and Au@HS-CNC (4.4 mol %) (Figure 4) showed absorbance bands around 2920 cm^−1^ due to the stretching vibration of the C–H bond in the HS–CH_2_–CH_2_–CH_2_-group. The peaks at 600–1180 cm^−1^ were designated to the ν_Si-O-Si_ and ν_Si-C-Si_ vibrations [36]. In comparison with the pure CNC, the HS-CNC and the Au@HS-CNC (4.4 mol %) catalysts exhibited an additional peak at 2546 cm^−1^ corresponding to the vibration of the HS-group [37]. However, the Au@HS-CNC (4.4 mol %) catalyst showed a weaker signal of the HS-group than the HS-CNC sample due to the coordination of the HS-ligand with the AuNPs. The solid-state ^13^C NMR spectra (Figure 5) further confirmed the presence of SH-groups in the Au@HS-CNC (4.4 mol %). In comparison with the pure CNCs, the Au@HS-CNC (4.4 mol %) catalyst clearly displayed two strong peaks at around 17 and 25 ppm owing to the C atoms connected with the S atoms in the HS–CH_2_–CH_2_–CH_2_ group [38]. The other peaks at around 65–80, and 105 ppm could be assigned to carbon atoms in the cellulose framework.

### Catalytic performances

The A^3^-coupling reaction of benzaldehyde, piperidine, and phenylacetylene was selected as the probe reaction to examine the catalytic activity of the Au@HS-CNC catalyst. Table 1 summarizes the catalytic performances of the catalyst with different Au-loadings, which were measured by inductively coupled plasma (ICP) analytical techniques. Both the HS-CNC and the Au sponge were inactive, implying that the Au is the active site and that the controlling of Au nanoparticle size is essential for the present reactions. The catalytic activity first increased with the increase of the Au loading up to 4.4 mol %. However, the activity slightly decreased with further increases in Au-loading up to 5.2 and 6.3 mol %. This decrease might be due to both the poor distribution of the Au active sites and the aggregation of the nanoparticles (See Figure S2, Supporting Information File 1). We determined the optimal Au-loading to be 4.4 mol %. Besides the Au-loading, we also investigated the effects of reaction solvents, temperature and reaction time on the catalytic efficiency. As shown in Table 1, one could conclude from the influence of the reaction time on the activity that the A^3^-coupling reaction reaches completion after 24 h under the present conditions. At a lower reaction temperature (25 °C), the Au@HS-CNC(4.4 mol %) showed lower conversion due to an incomplete reaction. We obtained the best conversion at a higher reaction temperature (above 80 °C). Solvent-free conditions proved to be the most effective for the A^3^-coupling reaction (Table 1, entry 23) and the conversion was comparable to that of the homogeneous catalyst (Table 1, entry 3). We obtained slightly lower conversions when using water or toluene as the solvent (Table 1, entries 14 and 15). Ethanol, acetonitrile, dichloromethane, tetrahydrofuran (THF), ethyl acetate (EA), dimethyl sulfoxide (DMSO), and *N*,*N*-dimethylformamide (DMF) afforded the products in moderate or low conversions (Table 1, entries 16–22). The optimized reaction conditions include 1.0 equiv of aldehyde, 1.2 equiv of amine, 1.5 equiv of alkyne, and 4.4 mol % of Au nanoparticles at 80 °C, solvent-free in air.

**Table 1:** Three-component coupling of benzaldehyde, piperidine, and phenylacetylene catalyzed by Au-based catalysts.^a^

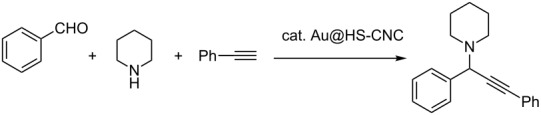			

entry	catalyst (mol %)	solvent/temp (°C)/time (h)	conversion (%)^b^

1	HS-CNC (0)	H_2_O/80/24	0
2	Au^0^ sponge (4.0)	H_2_O/80/24	0
3	HAuCl_4_ (1.0)	H_2_O/80/24	>99
4	Au@SH-CNC (2.9)	H_2_O/80/24	61
5	Au@SH-CNC (4.4)	H_2_O/80/24	87
6	Au@SH-CNC (5.2)	H_2_O/80/24	82
7	Au@SH-CNC (6.3)	H_2_O/80/24	81
8	Au@SH-CNC^c^(4.4)	H_2_O/80/24	67
9	Au@SH-CNC (4.4)	H_2_O/80/48	86
10	Au@SH-CNC (4.4)	H_2_O/80/12	73
11	Au@SH-CNC (4.4)	H_2_O/80/6	65
12	Au@SH-CNC (4.4)	H_2_O/120/24	85
13	Au@SH-CNC (4.4)	H_2_O/60/24	78
14	Au@SH-CNC (4.4)	H_2_O/rt/24	32
15	Au@SH-CNC (4.4)	toluene /80/24	92
16	Au@SH-CNC (4.4)	CH_2_Cl_2_/80/24	76
17	Au@SH-CNC (4.4)	ethanol/80/24	56
18	Au@SH-CNC (4.4)	MeCN/80/24	54
19	Au@SH-CNC (4.4)	DMSO/80/24	54
20	Au@SH-CNC (4.4)	THF/80/24	10
21	Au@SH-CNC (4.4)	DMF/80/24	trace
22	Au@SH-CNC (4.4)	EA/80/24	trace
23	Au@SH-CNC (4.4)	neat/80/24	100

^a^All reactions were carried out with benzaldehyde (0.2 mmol), piperidine (0.24 mmol), phenylacetylene (0.3 mmol), 0.2 mL solvent in a sealed well tube. ^b^Conversions were determined by ^1^H NMR of the crude reaction mixture. ^c^Catalyst was prepared by using a modified procedure reported by Tingaut et al.

To expand the scope of this A^3^-coupling, we used various aldehydes and amines as substrates under the optimized reaction conditions, and the results are summarized in Table 2. Both aromatic and aliphatic aldehydes provided the desired products in good to moderate yields (Table 2, entries 1–8). However, long chain aldehydes had a lower activity, giving lower yields (Table 1, entries 9, 10). We also observed good to moderate yields when the cyclic dialkylamines such as pyrrolidine, morpholine and azepane were used (Table 2, entries 11–19).

**Table 2:** Three-component coupling of aldehyde, amine, and phenylacetylene catalyzed by Au@SH-CNC catalysts in solvent-free conditions.^a^

entry	aldehyde	amine	product	yield (%)^b^

1	formaldehyde	piperidine	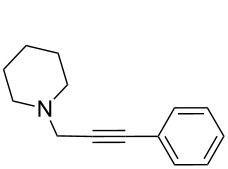	100
2	benzaldehyde	piperidine	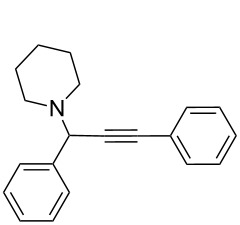	93
3	ethylbutyraldehyde	piperidine	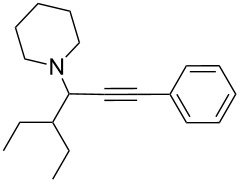	93
4	isobutyraldehyde	piperidine	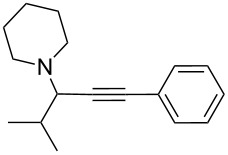	83
5	1-naphthaldehyde	piperidine	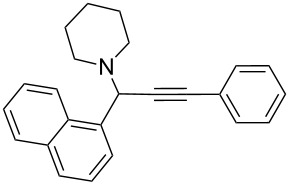	82
6	2-methylbutyraldehyde	piperidine	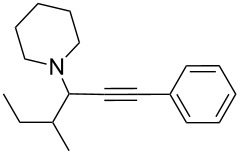	81
7	cyclohexanecarboxaldehyde	piperidine	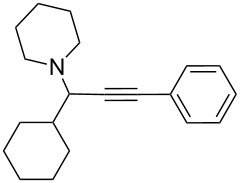	81
8	hydrocinnamaldehyde	piperidine	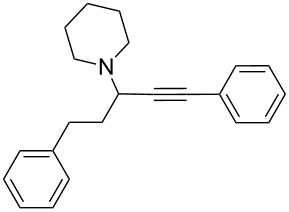	51
9	decanal	piperidine	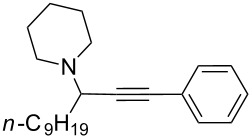	42
10	valeraldehyde	piperidine	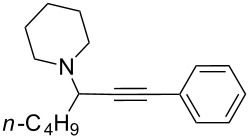	36
11	benzaldehyde	morpholine	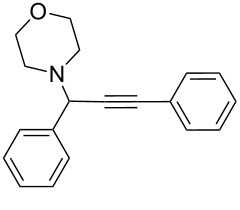	60
12	benzaldehyde	pyrrolidine	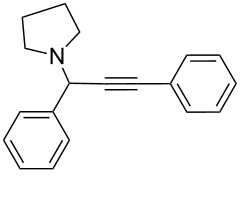	34
13	benzaldehyde	azepane	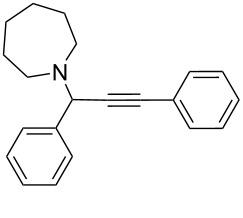	30
14	formaldehyde	morpholine	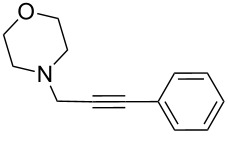	99
15	formaldehyde	pyrrolidine	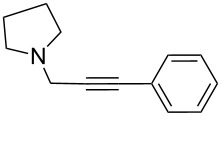	84
16	formaldehyde	azepane	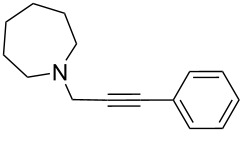	61
17	ethylbutyraldehyde	morpholine	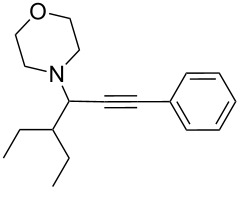	56
18	ethylbutyraldehyde	pyrrolidine	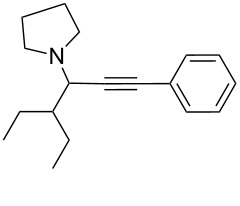	50
19	ethylbutyraldehyde	azepane	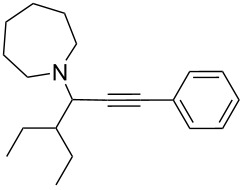	25

^a^All reactions were carried out with aldehyde (0.2 mmol), amine (0.24 mmol), phenylacetylene (0.3 mmol), and catalyst containing Au (4.4 mol %) in a sealed well tube, at 80 °C (oil bath) for 24 h. ^b^Yields were determined by ^1^H NMR of the crude reaction mixture.

### Catalyst recycling

**Figure 6:**
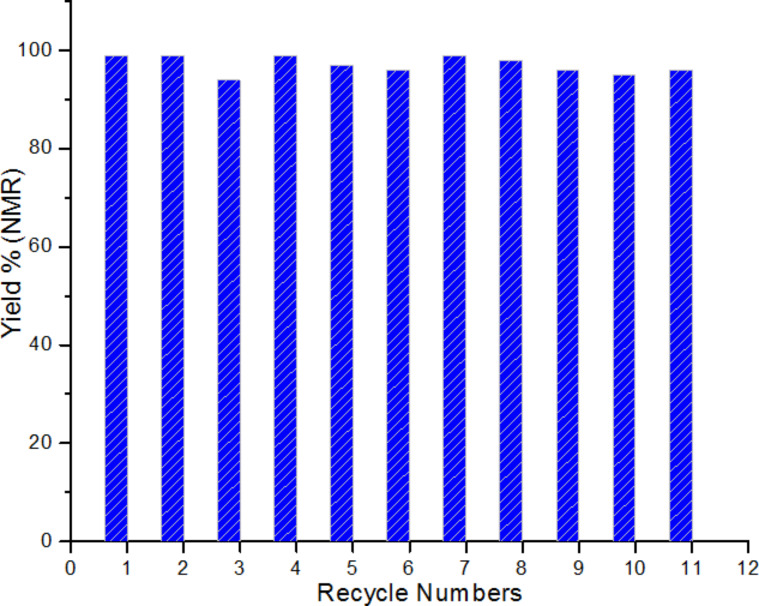
Recycling test of Au@HS-CNC (4.4 mol %) catalyst for the three-component coupling of formaldehyde, piperidine, and phenylacetylene (A^3^-coupling) under solvent-free conditions.

In order to determine the recycling ability of the catalysts, the following experiments were conducted. After completion of the reaction, the mixture was diluted with 0.5 mL deuterated chloroform (CDCl_3_) and filtered, and then the solid Au@HS-CNC(4.4 mol %) catalyst was washed 3 times with CDCl_3_, dried in vacuum, and then reused with a fresh charge of reactants for a subsequent run of reactions under identical conditions. Figure 6 demonstrates that the catalyst could be used repetitively more than 11 times without significant deactivation, suggesting its good reusability in solvent-free A^3^-coupling of formaldehyde, piperidine, and phenylacetylene. It is important to verify that the actual catalytic process is heterogeneous and not homogeneous [39]. For this reason, we did the following experiment: the solid catalyst was removed by filtering when the conversion was up to 45% in A^3^-coupling reactions, and then the solution reaction was continued under the same conditions. The conversion of the formaldehyde did not significantly increase, which strongly suggested that this catalytic process was a heterogeneous process.

## Conclusion

In summary, this work developed a new approach to design Au nanoparticles immobilized on the HS-functionalized CNCs. The novel Au@SH-CNC nanocomposite catalyst exhibited an excellent catalytic activity in the three-component coupling reaction of aldehyde-alkyne-amine (A^3^-coupling) either in water or without solvent, and could be used repetitively, which could reduce the cost and diminish the environmental impact of such reactions. Other immobilized metallic nanoparticle catalysts could also be designed based on the present method, which offered more opportunities for greener organic synthesis.

## Supporting Information

Detailed experimental procedures for the synthesis of CNCs and Au@HS-CNCs using a modified procedure reported by Tingaut et al. and the HRTEM images of the Au@HS-CNC (4.4 mol%) catalysts. TEM images of the (A) Au@HS-CNC (2.9 mol %), (B) Au@HS-CNC (5.2 mol %) and (C) Au@HS-CNC (6.3 mol %) catalysts and the analysis procedure of the product.

File 1 File Format PDF.
Experimental procedures, HRTEM images and analysis procedure.
