# Supplementary material for: A3-Coupling catalyzed by robust Au nanoparticles covalently bonded to HS-functionalized cellulose nanocrystalline films
Source: Beilstein J Org Chem. 2013 Jul 10;9:1388–96. doi: 10.3762/bjoc.9.155 (PMC3740685; doi:10.3762/bjoc.9.155)
Supplement: File 1 — File Format PDF. Experimental procedures, HRTEM images and analysis procedure. [file Beilstein_J_Org_Chem-09-1388-s001.pdf]

Supporting Information

for

A^3-Coupling catalyzed by robust Au nanoparticles covalently bonded to HS-functionalized cellulose nanocrystalline films

Jian-Lin Huang, Derek G. Gray*and Chao-Jun Li*

Department of Chemistry, McGill University, 801 Sherbrooke Street West, Montreal, Quebec H3A0B8, Canada

Email: Chao-Jun Li - cj.li@mcgill.ca; Derek G. Gray - derek.gray@mcgill.ca

*Corresponding author

Experimental procedures, HRTEM images and analysis procedure

Experimental methods

Materials:

Cotton from Whatman cellulose filter aid was used as the cellulose source. Deionized water (18.2 MΩ cm, Millipore Milli-Q Purification System) was used throughout. 3-Mercaptopropyltrimethoxysilane (MPTMS), $HAuCl_4$, solvents and other reagents were purchased from Sigma-Aldrich chemical company and were used without prior purification.

Preparation of CNCs

Suspensions of cellulose nanocrystals (CNCs) were prepared as previously described[1] by hydrolysis using 64% w/w sulfuric acid at 45 °C for 45 minutes under constant stirring. Typically, 20 g of cotton filter-aid was treated with 350 mL of acid. Immediately following the acid hydrolysis, the suspension was diluted 10-fold with deionized water to quench the reaction. The suspension was centrifuged at 10000 rpm for 6 minutes to concentrate the cellulose and to remove excess water and acid. The resultant precipitate was rinsed, re-centrifuged and dialyzed against water for 6 days until constant neutral pH was achieved in the effluent. Solid CNCs was also obtained by freeze-drying overnight or by air-drying.

Preparation of Au@HS-CNCs using a modified procedure reported by Tingaut et al[2].

In a standard experiment, a suspension consisting of 330 mg CNCs well-dispersed in an EtOH/H_2O (95/5, w/w) mixture (26.4 g) was stirred for 6 h at 25 °C. 3.96 g of 3-mercaptopropyltrimethoxysilane (MPTMS) was hydrolyzed in an EtOH/H_2O (95/5, w/w) mixture (35.6 g), reaching a final concentration of 10% (w/w). The pH was adjusted to 3.5 with AcOH and the solution was stirred at room temperature for 2 h. Then, the well-dispersed CNC suspension was added to this solution, and the mixture was diluted with EtOH/H_2O (95/5, w/w) to a final CNC concentration of 0.5% (w/w) with respect to the solvent. This suspension was stirred at room temperature for 4 h and filtered. Then, the wet HS-CNC cake was dried at 120 °C for 2 h, and was successively Soxhlet extracted with EtOH/H_2O (95/5, w/w) and dichloromethane for 4 h. The thiol-functionalized films were finally dried at 40 °C overnight. Finally, the HS-CNC films were added into $HAuCl_4$ ethanol solution and kept under stirring at room temperature for 24 h, and then filtered and dried at 40 °C overnight.

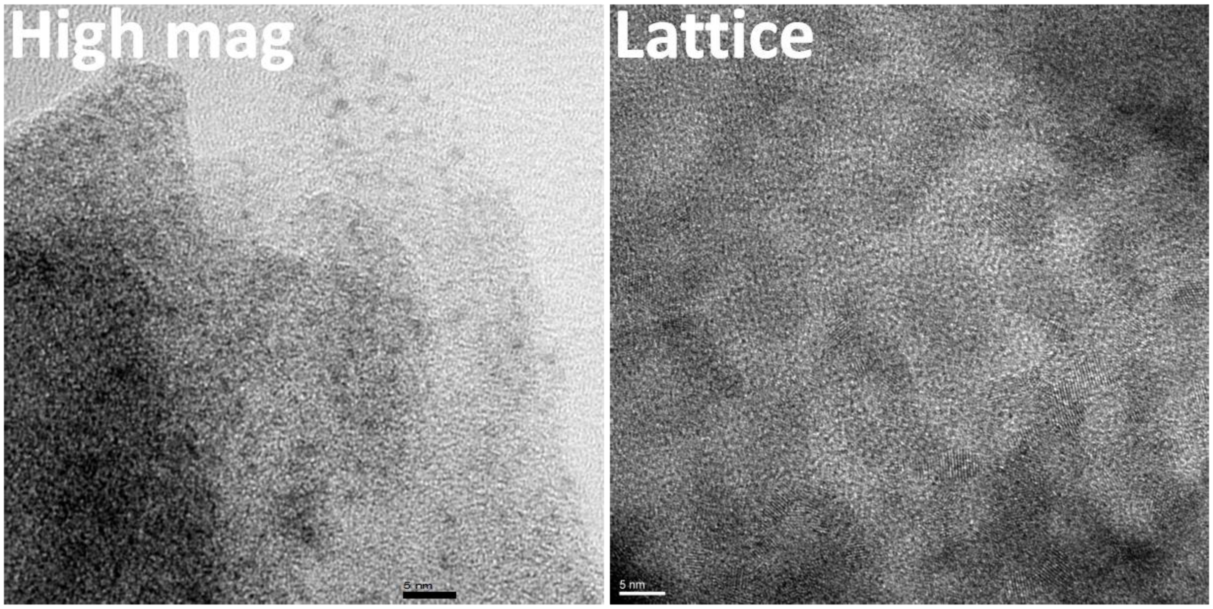

Figure S1: High-resolution TEM images of the Au@HS-CNC (4.4 mol %) catalyst.

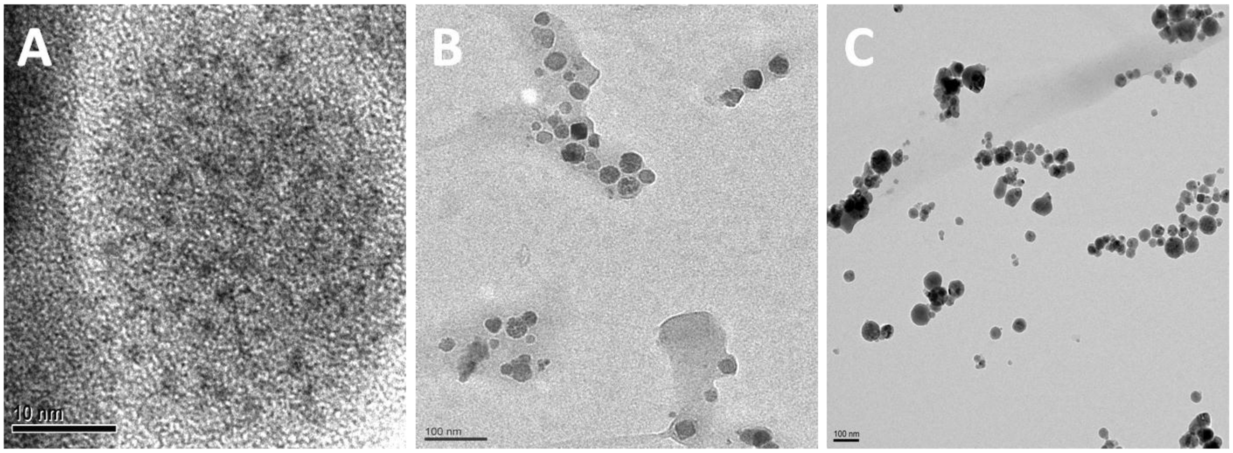

Figure S2: The TEM images of the (A) Au@HS-CNC (2.9 mol %), (B) Au@HS-CNC (5.2 mol %) and (c) Au@HS-CNC (6.3 mol %) catalyst.

The analysis procedure of the product:

After completion of the reaction, the mixture was diluted with 0.5 mL deuterated chloroform ($CDCl_3$), filtered and eluted through a short silica gel plug with more deuterated chloroform (2 x 0.5 mL), and dried over $MgSO_4$, then filtered and analyzed by 1H NMR. 1H NMR spectra were recorded on a Varian 400 MHz spectrometer and the chemical shifts were reported in parts per million (δ) relative to internal standard TMS (0 ppm) and all NMR spectra were recorded at room temperature.

References

1. (a) M. Hasani, E. D. Cranston, G. Westmana, D. G. Gray, *Soft Matter,* 2008, **4**, 2238-2244; (b) E. Kloser, D. G. Gray, *Langmuir,* 2010, **26**, 13450-13456.
2. P. Tingaut, R. Hauert, T. Zimmermann, *J. Mater. Chem.,* 2011, **21**, 16066-16076.
